# Unilateral pulmonary edema: a case report and review of the literature

**DOI:** 10.1186/s13256-018-1739-3

**Published:** 2018-08-14

**Authors:** Rangani Handagala, Udaya Ralapanawa, Thilak Jayalath

**Affiliations:** 1 Teaching Hospital, Peradeniya, Sri Lanka; 2 Department of Medicine, University of Peradeniya, Peradeniya, Sri Lanka

**Keywords:** Unilateral pulmonary edema, Mitral regurgitation, Brain natriuretic peptide, Heart failure

## Abstract

**Background:**

Unilateral pulmonary edema is an uncommon condition and is a rare clinical entity that is often misdiagnosed at the initial stages. In a majority of patients it occurs in the upper lobe of the right lung. There are many causes of unilateral pulmonary edema, but the commonest is the presence of a grade 3 mitral regurgitation. Due to its rare presentation, a high index of suspicion is required, and correct management is necessary to reduce the morbidity and mortality.

**Case presentation:**

We present a case of right-sided unilateral pulmonary edema in an 86-year-old Sinhalese Sri Lankan woman who presented with acute onset dyspnea with cardiogenic shock due to acute non-ST elevation myocardial infarction, complicated with grade 3 mitral regurgitation. She had clinical features of heart failure and pulmonary edema, but a chest X-ray showed unilateral infiltrates only on the right side. Distinguishing pneumonia from pulmonary edema according to chest X-ray findings was a challenge initially, and she was therefore initially treated for both conditions. She had remarkable clinical and radiological improvement after 12 hours of intravenously administered furosemide and glyceryl trinitrate therapy. Her brain natriuretic peptide level was elevated and further supported and confirmed the diagnosis retrospectively.

**Conclusions:**

Unilateral pulmonary edema is a completely reversible condition with good patient outcome if it is suspected early and treated early. Even in the absence of readily available echocardiogram skills, a clinical examination is of paramount importance in making a clinical decision in low-resource settings to reduce mortality.

## Background

Unilateral pulmonary edema (UPE) is uncommon, accounting for 2% of cardiogenic pulmonary edemas, and usually involves the upper lobe of the right lung [1]. The mechanism of UPE has been attributed to various causes [2]. Cardiogenic UPE is often misdiagnosed at first [1]. We present this case with UPE involving the entire right lung. Our patient had a grade 2 mitral regurgitation (MR) which she tolerated fairly well; however, she deteriorated into acute grade 3 MR following a non-ST elevation myocardial infarction (NSTEMI) which explains the importance of hemodynamics driving the cardiac filling pressures on an ischemic heart.

## Case presentation

An 86-year-old Sinhalese Sri Lankan woman who had been previously diagnosed as having hypertension, grade 2 MR, and ischemic heart disease with congestive cardiac failure, presented to our preliminary care unit with sudden onset shortness of breath at night while sleeping. She had eaten her dinner and taken her usual medications before sleeping. She had a New York Heart Association (NYHA) heart failure grade of class 2, and could manage her day-to-day activities without support. She could walk 25 meters and could climb 3–4 steps without becoming dyspneic. Apart from her usual symptoms she did not have fever, cough, or chest pain before admission. She is a housewife and mother of five children. She does not smoke tobacco or drink alcohol. At presentation she was on captopril 12.5 mg twice a day, atorvastatin 20 mg at night, soluble aspirin 75 mg at night, bisoprolol 2.5 mg once a day, and furosemide 40 mg in the morning.

On examination, she was found to be dyspneic, drowsy, pale, diaphoretic, and restless. Her body temperature was 37.0 °C. Her blood pressure (BP) was 90/60 mmHg, with a regular, low volume pulse rate of 102 beats per minute. Her heart sounds were unremarkable. Cardiac apex was not palpable. There was a pansystolic murmur at cardiac apex. Her respiratory rate was 26/minute. Her trachea was central and right-sided chest expansion was reduced. Bilateral crepitations and rhonchi were present more significantly on the right side. Her initial oxygen saturation checked by pulse-oximetry was 56% in room air. Her abdomen was not distended and there was mild right hypochondrial tenderness. There was no hepatosplenomegaly. Her cranial nerve examination was normal. Her limbs examination was normal with normal tone, power, and reflexes.

**Fig. 1:**
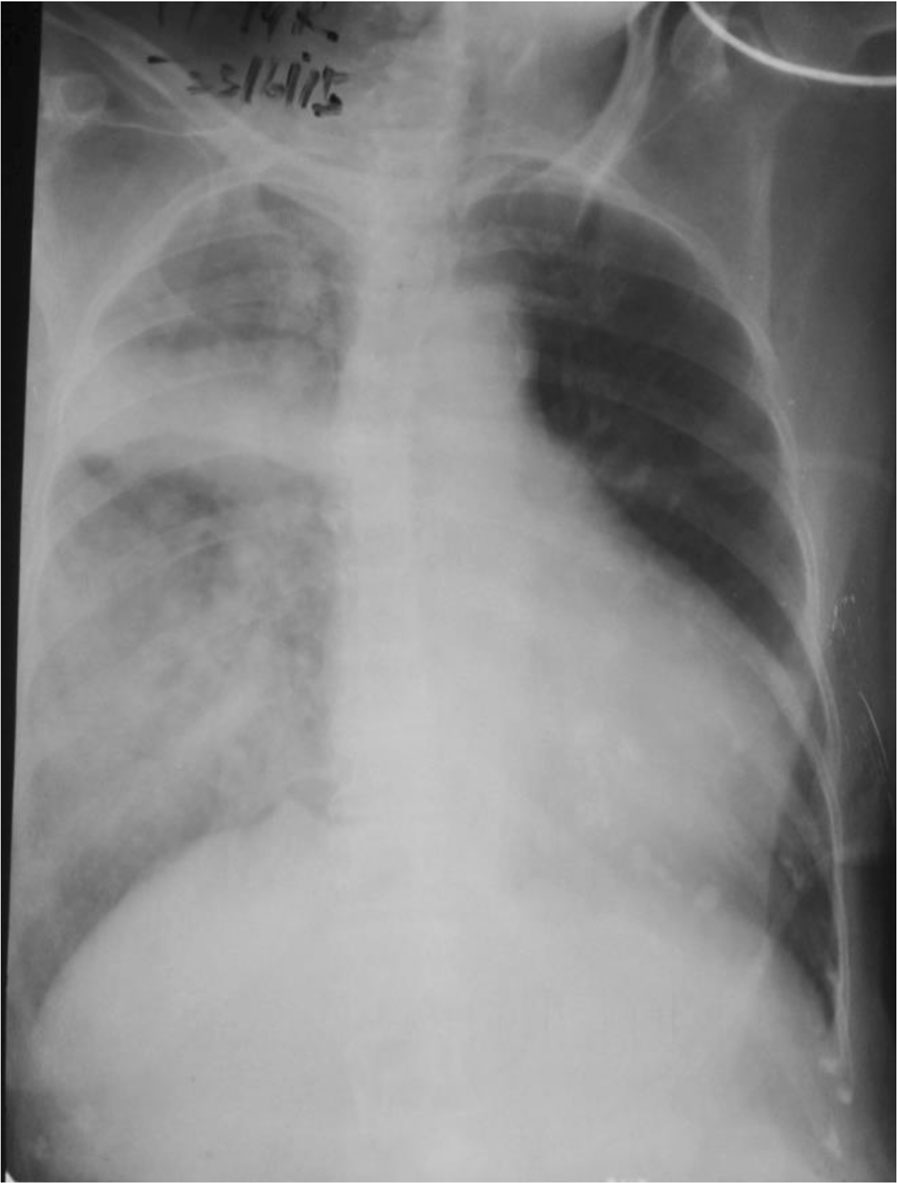
Chest X-ray on admission

An electrocardiogram showed ST depression in leads V5–V6 and poor R wave progression in leads V1–V4. Her chest X-ray revealed alveolar-interstitial infiltrates and a fluid collection around horizontal fissure in her right lung (Fig. 1). Laboratory tests showed a white blood cell count of 12,000/μL with 91.8% neutrophils, hemoglobin of 9.5 g/dL, packed cell volume of 30.3, mean corpuscular volume of 75 fl, mean corpuscular hemoglobin of 23.8 pg, mean corpuscular hemoglobin concentration of 31.4 g/dl, creatinine of 221.5 μmol/l, sodium level of 139 mEq/L, potassium level of 4.4 mEq/L, B-natriuretic peptide (BNP) of 2437.2 pg/ml (normal 450 for NYHA class 2), C-reactive protein (CRP) 7.56 mg/dL (< 10), and troponin I 59.2 ng/mL (< 0.01).

Although our patient’s temperature was normal, pneumonia could not be initially excluded in this older patient in the presence of a unilateral pulmonary infiltrate with effusion along the horizontal fissure, in combination with leukocytosis and awaiting CRP level (which took 4 hours to get the report), treatment with intravenously administered broad spectrum antibiotics (ceftriaxone 1 g twice a day and clarithromycin 500 mg twice a day) was initiated to cover severe community acquired pneumonia, and oseltamivir was started since there was an epidemic of influenza H1N1 at the time.

**Fig. 2:**
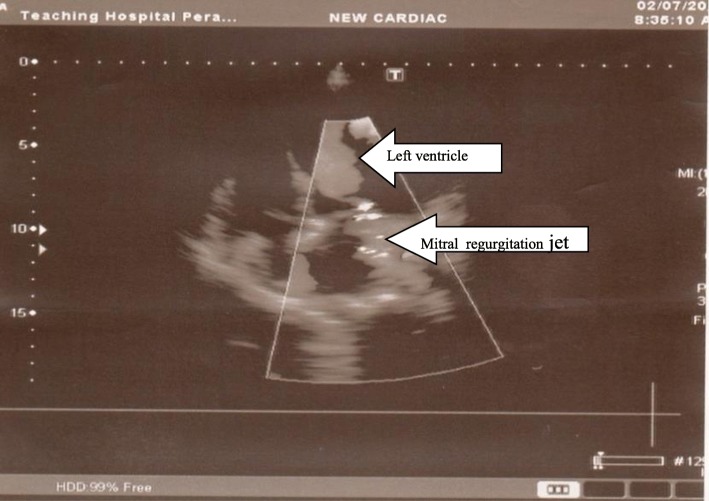
2D Echocardiogram showing mitral regurgitation

An emergency two-dimensional echocardiogram facility is not available in the preliminary care unit in our set up and our patient was not in a condition to be transferred to a place where a good quality echocardiogram machine was available to assess the severity of MR accurately. Echocardiography was done on third day of admission which disclosed: an ejection fraction of 25–30% with severe left ventricular (LV) dysfunction; and hypokinesia of anterior wall, LV apex, and lower 2/3 of interventricular septum, with an apical aneurysm. A two-dimensional echocardiogram showed grade 3 MR (Fig. 2). Although her BNP level was found to be high it took 4 days to get the report due to delays in laboratory processing. Therefore it helped us to support the diagnosis retrospectively.

**Fig. 3:**
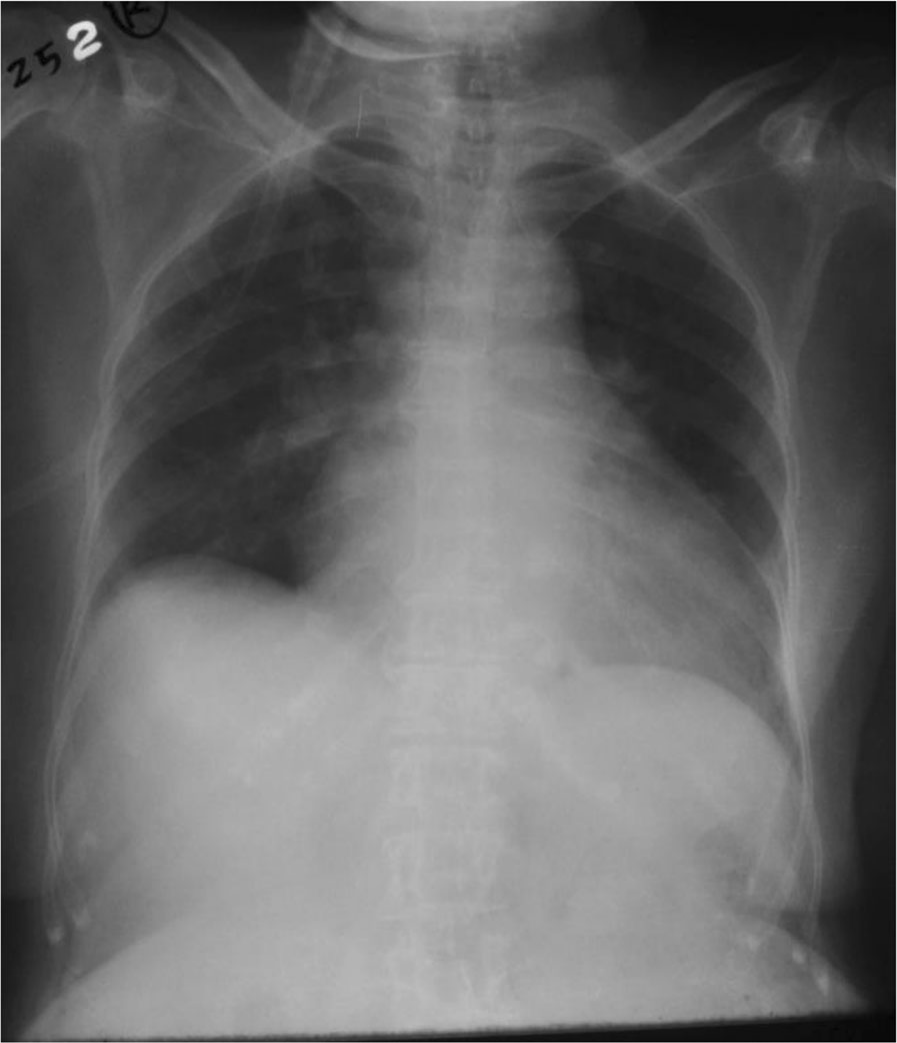
Chest X-ray taken after 12 hours. This X-ray was taken after intravenous furosemide/glyceryl trinitrate infusion

With all these challenges our patient was treated for severe acute on chronic heart failure although radiological evidence was unfavorable. Interestingly, she showed a remarkable improvement with preload reduction with loop diuretics and nitrates. After availability of troponin I levels she was treated for a NSTEMI on top of heart failure with intravenous heparin 500 units/hour infusion (her weight was 40 kg). Her condition was stabilized with adjustment of medical therapy for heart failure including diuretics, nitrates, and opioids. She had persistently low BP for which she needed inotropic support with dopamine and dobutamine which were tailed off subsequently. Repeat chest radiography taken 12 hours later showed complete resolution of the UPE (Fig. 3). Subsequently, her CRP was normal and antibiotics were de-escalated after 24 hours, but oseltamivir was continued. She had a fast and remarkable recovery to her preadmission state on day 5 of admission after which she was discharged. She refused any further cardiac intervention. Two weeks after discharge she was reviewed at a medical clinic and found to have NYHA class 2 heart failure. Her BP was120/80 mmHg and pulse rate was 70/minute. Her medications were uptitrated and she was followed up in a medical clinic. After 6 month she had a two-dimensional echocardiogram and revealed ejection fraction of 40% with grade 2–3 MR.

## Discussion

An 86-year-old woman previously diagnosed as having hypertension, grade 2 MR, and ischemic heart disease with congestive cardiac failure, presented with sudden onset shortness of breath. She had clinical features of heart failure and pulmonary edema, but a chest X-ray showed unilateral infiltrates only on the right side. Distinguishing pneumonia from pulmonary edema according to the chest X-ray findings was a challenge initially, and she was treated for both conditions. She had a remarkable clinical and radiological improvement after 12 hours of intravenously administered furosemide and glyceryl trinitrate therapy. Subsequently she was diagnosed as having NSTEMI, complicated with grade 3 MR. Her BNP level was elevated and further supported and confirmed the diagnosis of cardiogenic UPE retrospectively. UPE is a completely reversible condition with good patient outcome if it is suspected early and treated early. Even in the absence of readily available echocardiogram skills, a clinical examination is of paramount importance in making a clinical decision in low-resource settings to reduce mortality as discussed in this case report. Also this case highlights that acute cardiac ischemia can contribute to worsening a preexisting MR and precipitate UPE.

Acute cardiogenic pulmonary edema is a critical condition associated with high mortality, and may be caused by a variety of cardiac diseases, including coronary artery disease. The usual radiographic finding in acute cardiogenic pulmonary edema is bilateral symmetrical opacities in the central zones of the lungs, resulting in the classic “butterfly shadow” [3, 4]. UPE is a rare entity that can be mistaken for other causes of unilateral infiltrate on chest radiography, especially pneumonia. UPE has been reported after congestive heart failure, prolonged rest on one side in patients with cardiac decompensation or receiving large amounts of fluids, in cases of rapid expansion of the lungs after pleural effusion, and pneumothorax [5–7]. It is also seen in a normal lung in patients with unilateral pulmonary disease such as MacLeod syndrome and unilateral pulmonary artery hypoplasia or agenesis, pulmonary artery compression from aortic dissection or LV pseudo aneurysm, and pulmonary venous obstruction from mediastinal fibrosis [1]. However, it is mainly reported in association with severe MR [6].

Most cases of UPE associated with left-sided heart failure affect the right lung [4]. A possible explanation is the poorer lymphatic drainage of the right lung by the small-caliber right bronchomediastinal trunk in comparison with that of the left lung by the large-caliber thoracic duct. Another explanation relates to the left-sided cardiac enlargement that develops in most patients with heart failure and that may physically impede blood flow in the left pulmonary artery, thereby reducing capillary volume. However, severe MR remains the main cause of UPE [6]. An MR jet affecting predominantly the upper right pulmonary vein, can lead to a larger increase in mean capillary pressure on the right side and, consequently, a greater degree of right acute pulmonary edema. The main mechanism of MR in UPE is mitral leaflet prolapse, but functional MR may also be involved [6, 7].

During the early phase of acute myocardial infarction (AMI), transient ischemic MR is common and sometimes causes hemodynamic compromise. However, when several chordate tendineae or papillary muscle rupture occurs, this can lead to abrupt hemodynamic deterioration with cardiogenic shock. It is important to have a high index of suspicion for acute MR in any patient with acute pulmonary edema in the setting of AMI, especially if LV systolic function is well preserved [1]. Our patient did not have echocardiographic evidence of rupture of papillary muscles, but probably she had ischemia-induced papillary muscle dysfunction due to NSTEMI which would have pushed her to grade 3 MR during this admission causing right UPE. Table 1, which shows a summary of a recent literature review, highlights that almost all the patients with UPE have some degree of MR.

**Table 1:** Unilateral pulmonary edema: summary of recent literature review

Reference	Publication year	Demographics	Chest X-ray	Echocardiography	Underlying comorbidities
Kashiura *et al*. [9]	2017	Case 1: 72-year-old woman. Case 2: 40-year-old woman	Case 1: right-side limited alveolar-interstitial infiltrates with cardiomegaly. Case 2: left-side limited alveolar-interstitial infiltrates without cardiomegaly	Case 1: only sinus tachycardia. Case 2: mitral valve prolapse with severe regurgitation without left atrial dilation, and the regurgitant jet tended to blow toward the left side of the left atrium	Case 1: hypertension. Case 2: previously healthy
Mehta and Macduff [10]	2016	75-year-old woman	Right-sided pulmonary edema with sparing of the left lung	Flail anterior leaflet of the mitral valve with preserved left ventricular function	Out of hospital cardiac arrest following sudden breathlessness
Doshi and El Accaoui [11]	2016	75-year-old man	Asymmetric pulmonary edema with prominent vascular markings on the right lung	A flail anterior mitral leaflet secondary to ruptured posteromedial papillary muscle causing severe mitral regurgitation	Previously healthy
Venugopal *et al*. [12]	2015	18-year-old man	Unilateral pulmonary edema restricted to right side	Mild mitral regurgitation with no systolic or diastolic dysfunction	Previously healthy
Omran *et al*. [13]	2014	45-year-old woman	Mild cardiomegaly and left perihilar air space opacities	Not done	Chronic kidney disease, hypertension, and diabetes
Shin *et al*. [1]	2012	79-year-old man	Alveolar-interstitial infiltrates limited to the right lung	Ejection fraction of approximately 40% with global hypokinesia and mild mitral regurgitation	Current tobacco smoker and hypertension
Pandya *et al*. [3]	2012	74-year-old man	Right upper lobe infiltrates	Moderate pulmonary hypertension dilated left heart chambers, moderately severe mitral regurgitation, and ejection fraction of 20%.	Chronic obstructive airway disease (COPD), asbestosis-related pleural plaques, left lower limb deep vein thrombosis (DVT), and heavy alcohol consumption
Warraich *et al*. [14]	2011	52-year-old man	Right-sided infiltrates	Moderate aortic stenosis and 4 + mitral regurgitation, raising the possibility of a mitral valve perforation	Hypertensive, with a 40-pack-a-year smoking history
Gowrinath *et al*. [15]	2009	24-year-old man	Confluent alveolar opacities in the right mid and lower zones	Left ventricular hypertrophy and mild mitral regurgitation	Chronic kidney disease and hypertension
Peña *et al*. [16]	2005	76-year-old man	Acute pulmonary edema, predominantly right-sided	Ejection fraction of 50% with anteroapical akinesia and mild mitral regurgitation	Diabetes mellitus and hypertension
Mokta *et al*. [17]	2002	21-year-old man	Soft fluffy shadows in the left lung	Dilatation and systolic dysfunction of the right ventricle with normal left ventricular systolic function	Previously healthy
Lesieur *et al*. [18]	2000	Case1: 72-year-old man. Case 2: 75-year-old woman. Case 3: 90-year-old man	Case 1: interstitial infiltrate located only in the right lung without cardiomegaly. Case 2: right-sided pulmonary infiltrate. Case 3: interstitial infiltrate in the right upper lobe	Case 1: flail posterior leaflet of the mitral valve with grade 3/4 regurgitation and dilatation of the left atrium. Case 2: myxomatous mitral valve with a flail posterior leaflet and grade 4/4 regurgitation. Case 3: posterior buckling of the mitral valve with mitral regurgitation	Case 1: mitral valve prolapsed with grade 1 mitral regurgitation. Case 2: grade 2 mitral regurgitation. Case 3: mitral regurgitation, coronary artery disease, and atrial fibrillation

A unilateral radiography pattern may lead to a false diagnosis of pneumonia and so delay management. Although the induction of an acute phase reaction and an elevated peripheral leukocyte count, especially of neutrophils, in patients with AMI has been reported to be related to the extent of myocardial infarction and with prognosis, the association of unilateral pulmonary infiltrates with leukocytosis and/or acute respiratory distress often leads to antibiotic therapy, despite the absence of fever, especially in older patients [1]. Furthermore, patients with UPE present with a higher risk of death than patients with bilateral pulmonary edema, and delay in adequate treatment of UPE may be one explanation for this increased mortality [1]. The absence of fever, a history of sudden onset of dyspnea, and elevated levels of BNP, may help to differentiate UPE from other diagnoses. However, in our case BNP levels helped us to diagnose retrospectively. A murmur on examination can be useful, especially for organic MR. However, the intensity of the murmur is low and correlates poorly with the degree of regurgitation in acute ischemic MR. Echocardiography is useful in determining the severity of MR and its cause. The availability of a good quality echocardiogram on admission would have reduced the morbidity further and improved patient outcome and reduced the hospital stay [7]. Cardiogenic UPE can easily be mistaken for pneumonia or some other pulmonary pathological condition. The availability of correct resources at an appropriate time would make a dramatic difference in management. Since we cannot guarantee the reliability of supplementary medical backup in our set up, parameters such as physical examination findings and the clinical course should also be taken into account. Where time factor decides the mortality, skills in clinical examination are of paramount importance in making a clinical decision in a low-resource setting to reduce mortality.

In this case, the patient was treated empirically for both pulmonary edema and chest infection and subsequent de-escalation of antibiotics. Cardiogenic shock developed due to NSTEMI on top of a weak heart. Our patient was a diagnosed case of grade 2 MR, but it is doubtful that grade 2 MR would be a cause of pulmonary edema. Acute myocardial ischemia could exacerbate diastolic dysfunction with afterload mismatch and possibly the reduced systolic function, as well as transient ischemic MR due to papillary muscle dysfunction, may cause grade 3 MR, which was seen in this admission [1]. When regurgitant volume increases suddenly due to transient ischemic MR, the acute rise in left atrial (LA) pressure can be transmitted back to the pulmonary circulation, generating pulmonary edema. UPE can occur not only because the direction of the MR jet affects predominantly the upper right pulmonary vein, but also because of differences between the mechanisms controlling tissue osmotic pressure in the two sides of the lung. For example, in the case of a sudden increase of LA pressure, the right lung parenchyma can develop edema due to the low excretory capability of its lymphatic drainage compared to that of the left lung, precipitating an event of right-sided UPE [1, 8]. Recovery of the ischemic papillary muscle and the limited number of episodes of LA hypertension, which are the main determinants of pulmonary edema after AMI, may have contributed to the resolution of the pulmonary edema with treatment over a day. The lesson of this case is that it is important to acknowledge that AMI can present as UPE, even in patients without severe MR or any preexisting pulmonary disease affecting the vasculature or parenchyma of a lung.

The most important take home message from this case is that even with minimal resources available one can save a life in a critical situation if one’s clinical suspicion and diagnosis overrides the supplementary evidence.

## Conclusion

UPE is a completely reversible condition with good patient outcome if it is suspected early and treated early.

## Abbreviations

AMI: Acute myocardial infarction
BNP: B-natriuretic peptide
BP: Blood pressure
CRP: C-reactive protein
LA: Left atrial
LV: Left ventricular
MR: Mitral regurgitation
NSTEMI: Non-ST elevation myocardial infarction
NYHA: New York Heart Association
UPE: Unilateral pulmonary edema

## Authors’ information

RH (MBBS, Dep. Crit. Care Med) is a registrar in critical care medicine, Teaching Hospital, Peradeniya, Sri Lanka. UR (MBBS, MD, MRCP, FRCP) is a Senior Lecturer and Consultant Physician, Department of Medicine, University of Peradeniya, Sri Lanka. TJ (MBBS, MD, MRCP, FRCP, FRCPE, FACP, FCCP) is a Professor in Medicine and Consultant Physician, Department of Medicine, University of Peradeniya, Sri Lanka.
